# Supplementary material for: Terminal α1,2-fucosylation of glycosphingolipids by FUT1 is a key regulator in early cell-fate decisions
Source: EMBO Rep. 2024 Sep 10;25(10):4433–64. doi: 10.1038/s44319-024-00243-1 (PMC11467398; doi:10.1038/s44319-024-00243-1)
Supplement: Supplementary file 1 — Appendix [file 44319_2024_243_MOESM1_ESM.pdf]

Appendix

Appendix Figure S1 (related to Figure 1) Co-expression of FUT1 with NE markers and FUT2 and SEC1 gene expression in early embryonic development……………………….1

Appendix Figure S2 (related to Figure 2) GT gene expression is changed during pluripotency and 14 d after hESC differentiation into CMs...3

Appendix Figure S3 (related to Figure 3) The expression of FUT1 and α-fucose in iPSCs during pluripotency and differentiation is identical to that of hESCs…………………….4

Appendix Figure S4 (related to Figure 4) Silencing FUT1 alters mesoderm gene expression………………………………………………………………………………..5

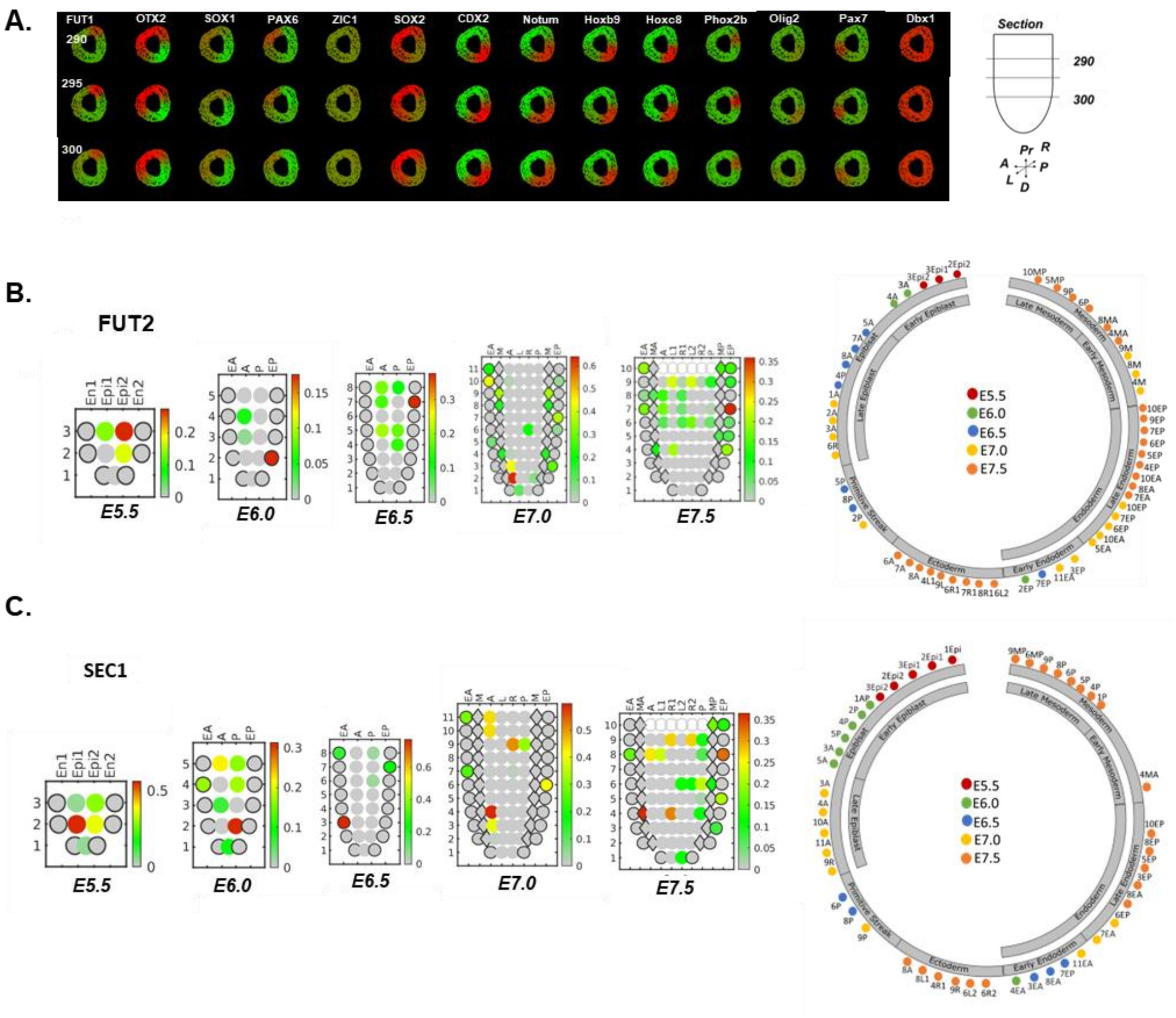

Appendix Figure S1 (related to Figure 1)

Co-expression of FUT1 with NE markers and FUT2 and SEC1 gene expression in early embryonic development

(A) Left: Illustration of FUT1 gene and NE markers in E7.5 embryos showing FUT1 gene co-expression with OTX2, PAX6, ZIC1, SOX2, SOX1, HOXC8, PHOX2B, OLIG2, PAX7, HOXB9, and DBX1 in sections 295–300, using *in silico* transcriptomic analysis in 3D and employing the database of mouse gastrulation on E5.5–E7.5. FUT1 is co-expressed with SOX2 (EPI), OTX2, and DBX1; NE markers of fore-midbrain and intermediate neural tube. Right: Schematic showing embryo sections for transcriptomic analysis. A, anterior; P, posterior; L, left; R, right; Pr, proximal; D, distal. (B) Left: 2D corn plots showing the spatiotemporal expression of the FUT2 gene in E5.5–E7.5 embryos. Right: Circle diagrams demonstrating FUT2 gene expression base on tissue classification. (C) Left: 2D corn plots showing the spatiotemporal expression of SEC1 in E5.5–E7.5 embryos. Right: Circle diagrams demonstrating SEC1 gene expression based on tissue classification. NE, neuroectoderm; En1

and En2, divided endoderm; Epi1 and Epi2, divided epiblast; EA, anterior endoderm; EP, posterior endoderm; A, anterior; P, posterior; M, whole mesoderm; L, left lateral; R, right lateral; L1, anterior left lateral; R1, anterior right lateral; L2, posterior left lateral; R2, posterior right lateral; MA, anterior mesoderm; MP, posterior mesoderm.

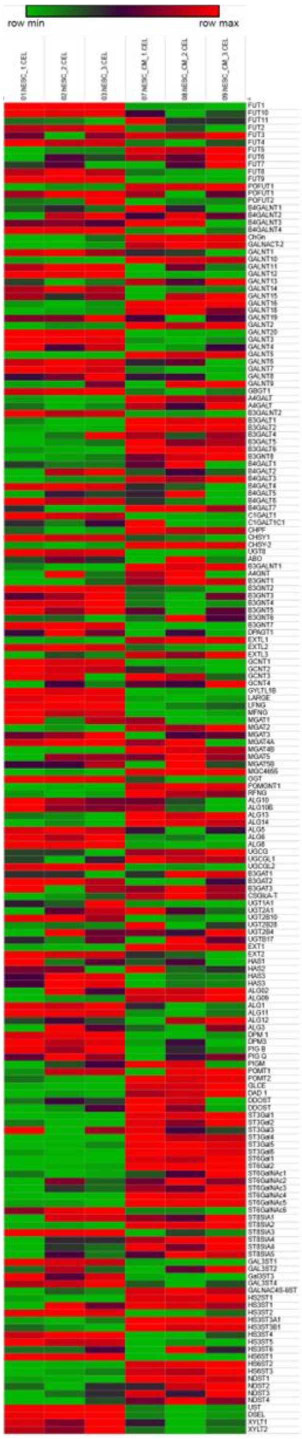

Appendix Figure S2 (related to Figure 2)

GT gene expression is changed during pluripotency and 14 d after hESC differentiation into CMs

Heatmap comparing the expression level of 181 genes involved in glycosylation of hESCs and hESC-derived CMs after 14 d of differentiation. n = 3 technical replicates. Data are means ± SEM. $p < 0.001$ (Two-tailed unpaired t test).

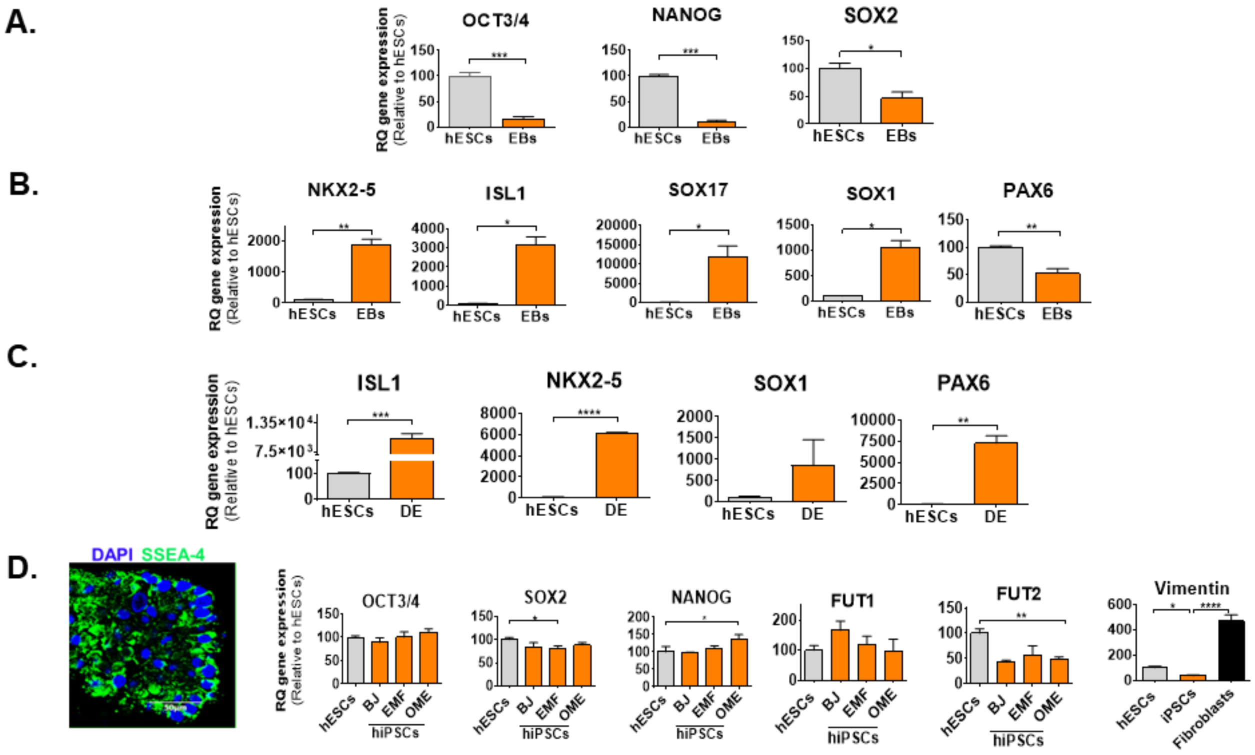

Appendix Figure S3 (related to Figure 3)

The expression of FUT1 and α-fucose in iPSCs during pluripotency and differentiation is identical to that of hESCs

(A) Quantification of the gene expression of pluripotent markers, OCT3/4, SOX2, and NANOG in hESCs and day 5 EBs, as measured by qPCR. n = 3 technical replicates. (B) Quantification of the mRNA expression of tri-germ layer markers, NKX2.5, ISL1, SOX17, SOX1, and PAX6 in WT hESCs and day 5 EBs. n = 3 technical replicates. (C) Quantification of DE gene expression in WT hESCs and day 8 DE. n = 3 technical replicates. Data are means ± SEM. NS>0.05, $^{*}p < 0.05$, $^{**}p < 0.01$, $^{***}p < 0.001$, $^{****}p < 0.0001$ (Two-tailed unpaired t tests). (D) Left: Representative images of hiPSCs growing on Matrigel showing SSEA-4 (green) and DAPI (blue) for nucleus. Scale bars represent 50 μm. Right: Quantification of the pluripotent gene expression, OCT3/4, SOX2, and NANOG, and of FUT1 and FUT2 in hESCs in three lines of hiPSCs (BJ, EMF and OME), and Vimentin in hESCs, a pool of hiPSCs, and a pool of human fibroblasts before reprogramming. Pooled sample, n = 3 cell lines. n = 3

technical replicates. Data are means ± SEM. NS>0.05 *p < 0.05, **p < 0.01, ***p < 0.001, ****p < 0.0001 (Ordinary one-way ANOVA).

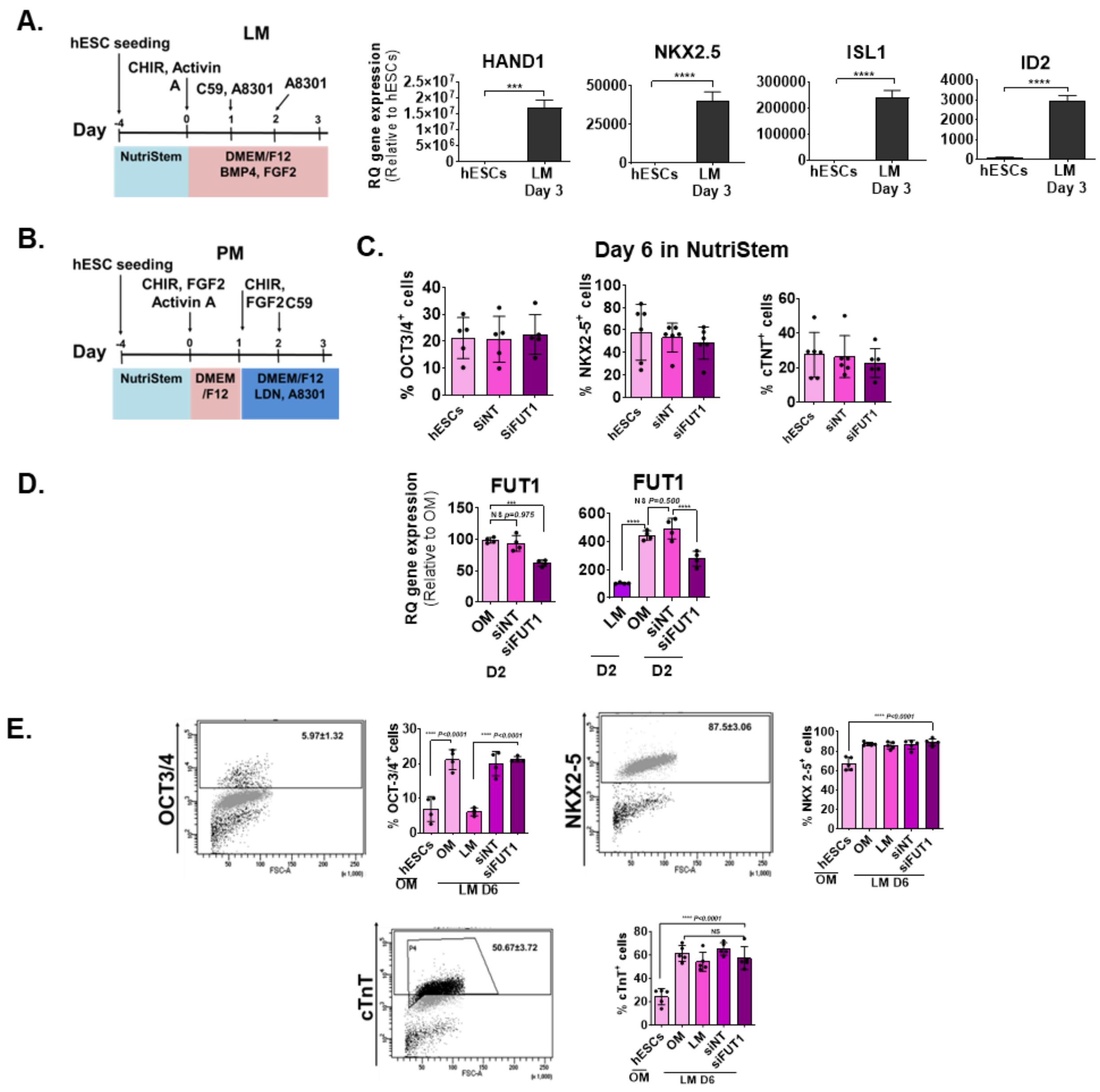

Appendix Figure S4 (related to Figure 4)

Silencing FUT1 alters mesoderm gene expression

(A) Top left: Schematic showing hESC differentiation into LM using Wnt/FGF/BMP and Nodal signaling over 3 d. Top right: Quantification of the gene expression of LM, HAND1, NKX2.5, and ISL1 and ID2 in WA09 hESCs and 3 d hESC-derived LM. n=3 technical replicates. (B) Schematic showing hESC differentiation into PM using Wnt/FGF/BMP and

Nodal signaling over 3 d. (C) Quantification of the percent of positive cells expressing OCT3/4, NKX2.5, and cTnT in WT, siNT, and siFUT1, WA09 hESCs grown for 6 d in NutriStem. n=3 technical replicates. (D) Left: Quantification of FUT1 transcripts in WA09 hESCs grown in OM and in siNT and siFUT1, WA09 hESCs grown for 2 d in NutriStem. Right: Quantification of the gene expression of FUT1 in WA09 hESCs grown in OM and 2 d after differentiation into LM compared to the gene expression of FUT1 in siNT and siFUT1, WA09 hESCs 2 d after differentiation into LM. n=4 biological replicates. (E) Quantification of the percent of positive cells for pluripotent marker, OCT3/4 and for cardiac-specific markers, NKX2.5 and cTnT in WT hESCs grown in NutriStem or OM in NutriStem medium, and after differentiation into CM for 6 d in WT, siNT and siFUT1 hESCs. H9.1 hESCs were silenced with a mixture of 3 siRNA for FUT1 and 1 NT siRNA. Error bars represent ± SDs for n=3 technical replicates. The housekeeping gene GAPDH was used for normalization. Data presented are relative to the values of day 0 WT or siNT hESCs. Data are means ± SEM. *$p < 0.05$, **$p < 0.01$, ***$p < 0.001$, ****$p < 0.0001$ (two-tailed unpaired Student's t-tests).
