# Supplementary material for: Terminal α1,2-fucosylation of glycosphingolipids by FUT1 is a key regulator in early cell-fate decisions
Source: EMBO Rep. 2024 Sep 10;25(10):4433–64. doi: 10.1038/s44319-024-00243-1 (PMC11467398; doi:10.1038/s44319-024-00243-1)
Supplement: Supplementary file 5 — Expanded View Figures [file 44319_2024_243_MOESM5_ESM.pdf]

Expanded View Figures

Figure EV1. Co-expression of FUT1 with pluripotency and primary germ layer markers in early embryonic development.

(**A**) Left: Representative in silico 3D transcriptomic analysis showing co-expression of FUT1 with pluripotent markers (NANOG, SOX2, and OCT3/4) on E6.0 in sections #351–370 from a sequential series of sections. Right: 2D corn plots showing FUT1 and pluripotent gene expression on E6.0. Abbreviations represent A, anterior; P, posterior; L, left; R, right; Pr, proximal; D, distal. (**B**) 2D corn plots showing the expression of ectoderm markers, OTX2, PAX6, ZIC1, SOX2, SOX1, HOXC8, PHOX2b, OLIG2, PAX7, HOXB9, and DBX1 on E7.5 embryos. (**C**) 2D corn plots showing the expression of DE markers, HHEX, FOXA2, SOX17, and FOXA1 on E7.5. (**D**) 2D corn plots showing the spatiotemporal expression mesoderm genes, BRY (T gene) and MIXL1 on E7.0–E7.5; HOPX, NKX2.5, ISL1, HAND1, IRX3, MESP2, FOXC1, FOXF1, and CDX2 on E7.5. 2D and 3D gene expression analysis was obtained by using the database of mouse gastrulation on E5.5–E7.5 (http://egastrulation.sibcb.ac.cn).

 © The Author(s)

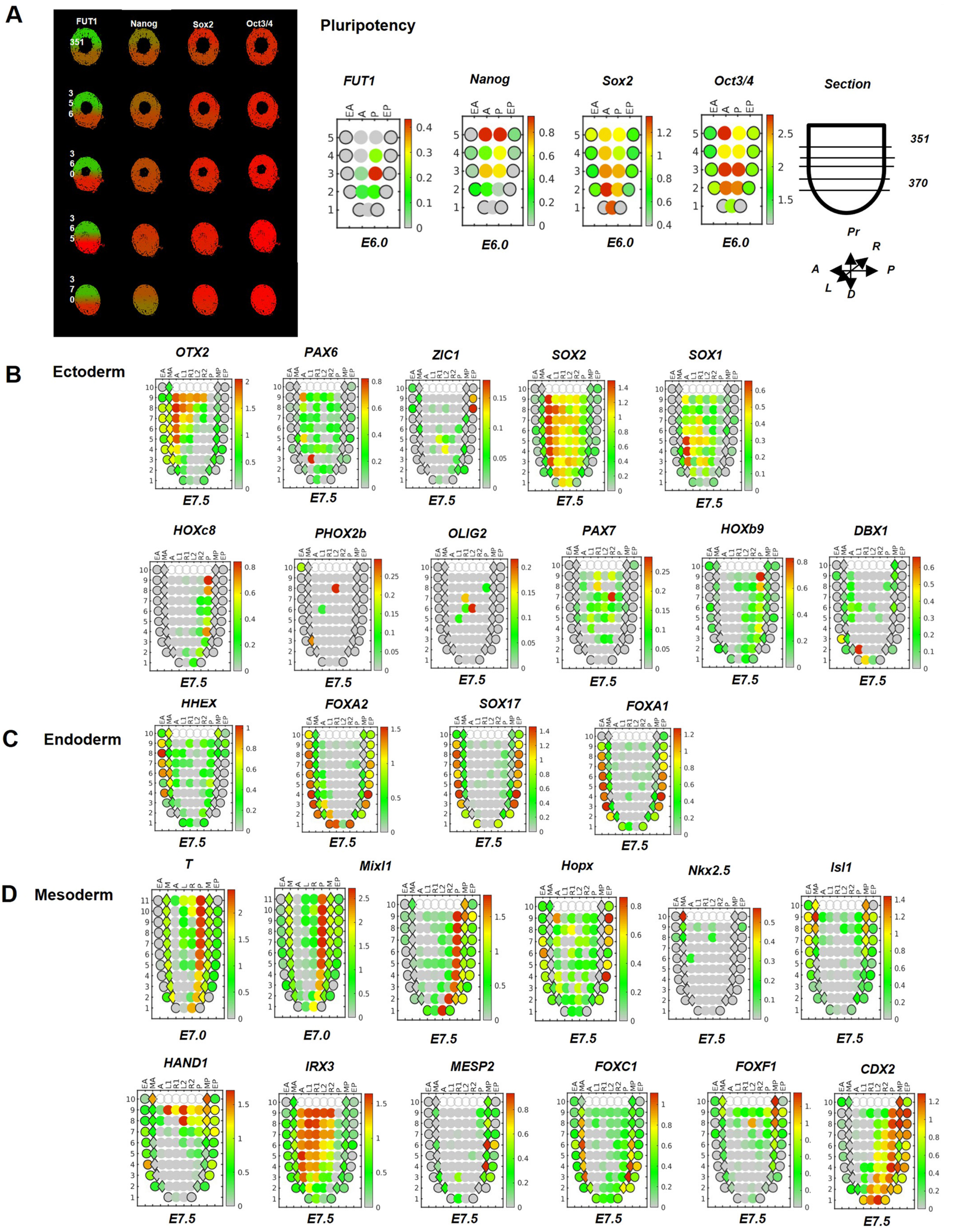
A
FUT1
Nanog
Sox2
Oct3/4
351
356
360
365
370
Pluripotency
FUT1
Nanog
Sox2
Oct3/4
Section
E6.0
351
370
Pr
R
A
P
L
D
B
Ectoderm
OTX2
PAX6
ZIC1
SOX2
SOX1
HOXc8
PHOX2b
OLIG2
PAX7
HOXb9
DBX1
E7.5
C
Endoderm
HHEX
FOXA2
SOX17
FOXA1
D
Mesoderm
T
Mixl1
Hopx
Nkx2.5
Isl1
E7.0
HAND1
IRX3
MESP2
FOXC1
FOXF1
CDX2

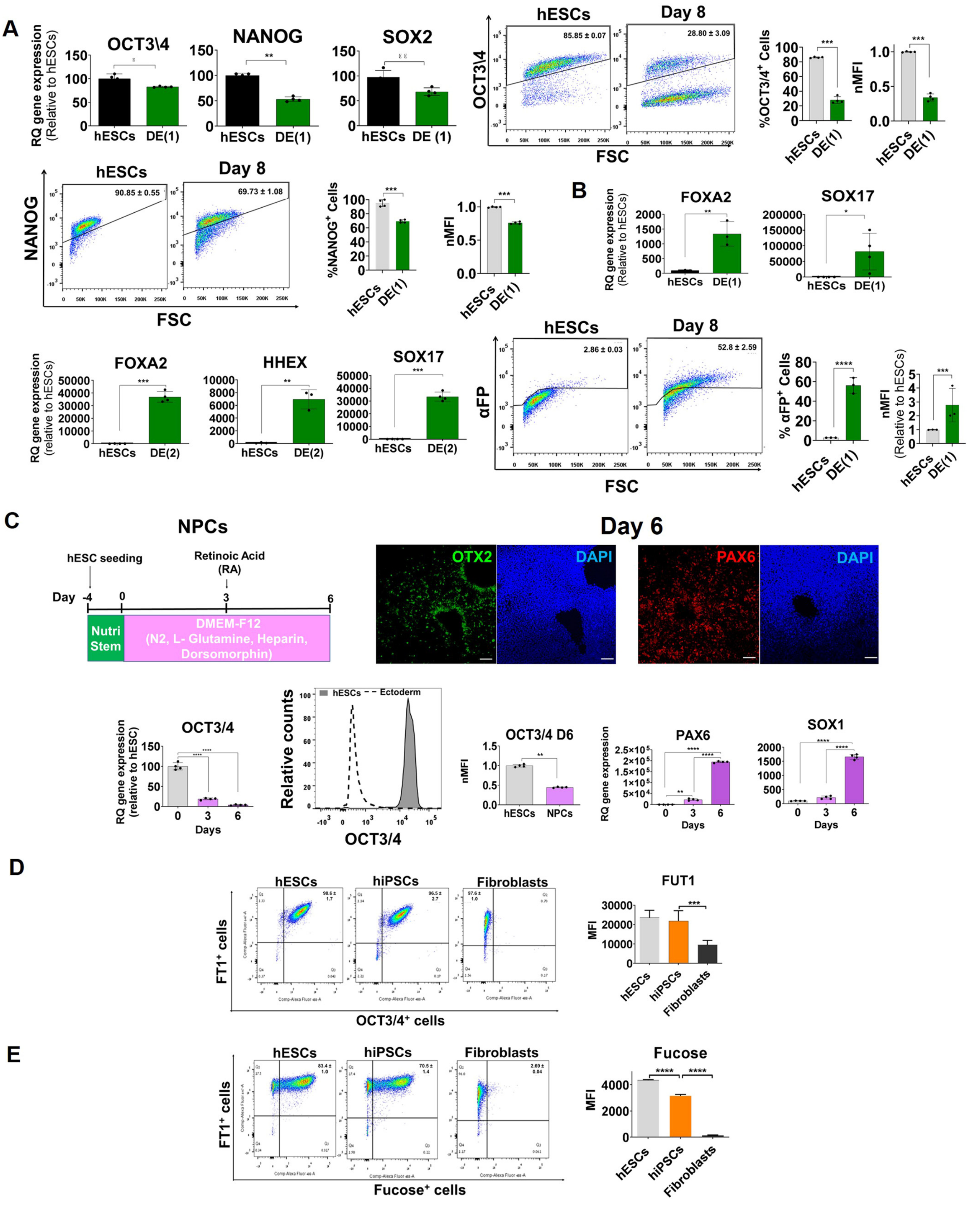
A
OCT3\4
NANOG
SOX2
RQ gene expression (Relative to hESCs)
hESCs DE(1)
hESCs
Day 8
85.85 ± 0.07
28.80 ± 3.09
OCT3\4
FSC
%OCT3/4+ Cells
nMFI
90.85 ± 0.55
69.73 ± 1.08
%NANOG+ Cells
B
FOXA2
SOX17
HHEX
hESCs DE(2)
αFP
2.86 ± 0.03
52.8 ± 2.59
% αFP+ Cells
nMFI (Relative to hESCs)
C
NPCs
Day 6
hESC seeding
Retinoic Acid (RA)
Day -4 0 3 6
Nutri Stem
DMEM-F12 (N2, L- Glutamine, Heparin, Dorsomorphin)
OTX2
DAPI
PAX6
OCT3/4
RQ gene expression (relative to hESC)
Days
Relative counts
hESCs
Ectoderm
OCT3/4 D6
hESCs NPCs
PAX6
RQ gene expression
SOX1
D
hESCs
hiPSCs
Fibroblasts
98.6 ± 1.7
96.5 ± 2.7
97.6 ± 1.0
FT1+ cells
OCT3/4+ cells
FUT1
MFI
E
83.4 ± 1.0
70.5 ± 1.4
2.69 ± 0.04
Fucose+ cells
Fucose

 © The Author(s)

Figure EV2. FUT1 is downregulated during PC differentiation into the tri-germ layer lineages.

(**A**) Up at left: Quantification of pluripotent gene expression as measured by qPCR. Up at right: Quantification of the percentage of hESCs and hESCs-derived DE on day 8 showing the expression of OCT3/4 in histograms, bar chart, and MFI relative to hESCs. Bottom: Quantification of the percentage of hESCs and hESCs-derived DE on day 8 showing the expression of NANOG in histograms, bar chart, and MFI relative to hESCs. $n = 4$ technical replicates. (**B**) Up and bottom at left: Quantification of DE-specific markers, FOXA2 and SOX17 as measured by qPCR after hESC differentiation into DE using protocol 1, and FOXA2, SOX17, and HHEX as measured by qPCR after hESC differentiation into DE using protocol 2 ($n = 3$ technical replicates). Bottom at right: Quantification of the percentage of hESCs and hESCs-derived DE on day 8 showing the expression of α-FP in histograms and bar chart and MFI relative to hESCs. (**C**) Up at left: Schematic illustration of hESC differentiation into NPCs via bFGF and RA signaling and BMP inhibition over 6 d. Up at right: Representative immunofluorescent cultures illustrate day 6 NPCs expressing OTX2 (green) and PAX6 (red). Nuclei are stained with DAPI (blue). Scale bars represent 100 µm. Bottom: qRT-PCR and flow cytometry analyses of OCT3/4, PAX6, SOX1. The expression of FUT1 and α-fucose in iPSCs during pluripotency and differentiation is identical to that of hESCs (**D**) Quantification of the percent of positive cells expressing both the FT1 and OCT3/4 proteins and MFI in hESCs, a pool of hiPSCs and a pool of human fibroblasts. Pooled sample, $n = 3$ cell lines. $n = 3$ technical replicates. (**E**) Quantification of the percent of positive cells expressing FT1 protein and α-fucose residues and MFI in hESCs, a pool of hiPSCs, and a pool of human fibroblasts. Pooled sample, $n = 3$ cell lines. $n = 3$ technical replicates. Data information: In qPCR (**A**, **B**), data are presented as means ± SD. Two-tailed Student's t-tests *$p < 0.05$, **$p < 0.01$, ***$p < 0.001$, ****$p < 0.0001$. In FACS (**A–E**), data are presented as means ± SD. Ordinary One-way ANOVA **$p < 0.01$, ***$p < 0.001$, ****$p < 0.0001$.

© The Author(s)

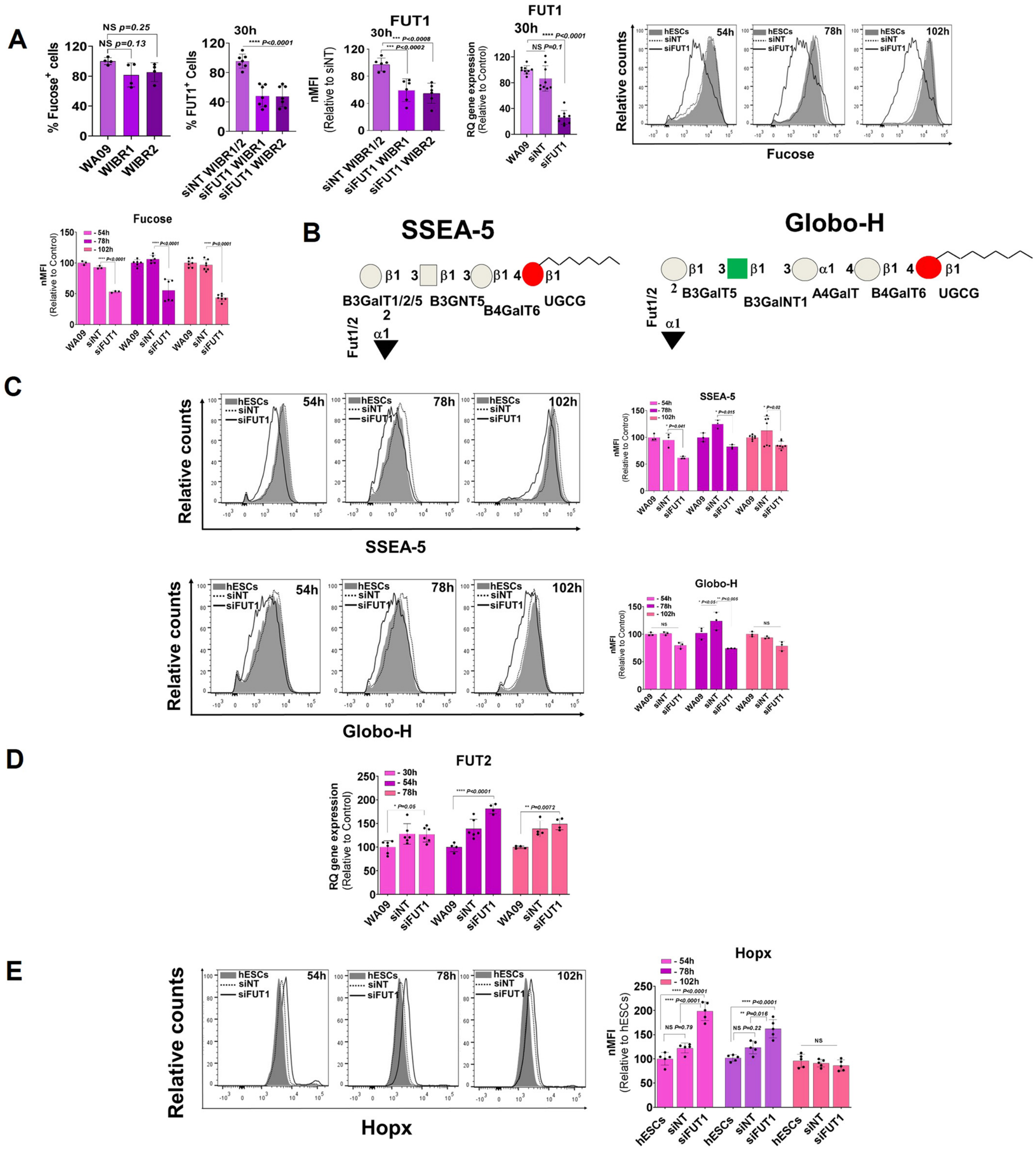
A
NS p=0.25
NS p=0.13
% Fucose+ cells
WA09
WIBR1
WIBR2
30h
**** P<0.0001
% FUT1+ Cells
siNT WIBR1/2
siFUT1 WIBR1
siFUT1 WIBR2
FUT1
30h
*** P<0.0008
*** P<0.0002
nMFI
(Relative to siNT)
FUT1
30h
**** P<0.0001
NS P=0.1
RQ gene expression
(Relative to Control)
WA09
siNT
siFUT1
Relative counts
hESCs
siNT
siFUT1
54h
78h
102h
Fucose
Fucose
- 54h
- 78h
- 102h
nMFI
(Relative to Control)
**** P<0.0001
B
SSEA-5
β1
3
β1
3
β1
4
β1
B3GalT1/2/5
B3GNT5
B4GalT6
UGCG
2
α1
Fut1/2
Globo-H
β1
3
β1
3
α1
4
β1
4
β1
2
B3GalT5
B3GalNT1
A4GalT
B4GalT6
UGCG
Fut1/2
α1
C
Relative counts
hESCs
siNT
siFUT1
54h
78h
102h
SSEA-5
SSEA-5
- 54h
- 78h
- 102h
* P=0.041
* P=0.015
* P=0.02
nMFI
(Relative to Control)
Relative counts
hESCs
siNT
siFUT1
54h
78h
102h
Globo-H
Globo-H
- 54h
- 78h
- 102h
NS
* P<0.05
** P=0.005
NS
nMFI
(Relative to Control)
WA09
siNT
siFUT1
D
FUT2
- 30h
- 54h
- 78h
* P=0.05
**** P<0.0001
** P=0.0072
RQ gene expression
(Relative to Control)
WA09
siNT
siFUT1
E
Relative counts
hESCs
siNT
siFUT1
54h
78h
102h
Hopx
Hopx
- 54h
- 78h
- 102h
**** P<0.0001
**** P<0.0001
NS P=0.79
**** P<0.0001
** P=0.016
NS P=0.22
NS
nMFI
(Relative to hESCs)
hESCs
siNT
siFUT1

 © The Author(s)

Figure EV3. Silencing of FUT1 alters mesoderm gene expression.

(**A**) Top at left: Quantification of the percent of positive cells expressing α-fucose residues during pluripotency in three lines of hESCs: WA09, WIBR1, and WIBR2, as measured in FACS. Top at center: Quantification of the percent of positive cells expressing FT1 protein and MFI of WIBR1 and WIBR2 hESCs 30 h after FUT1 mRNA silencing (siFUT1). Negative control is WIBR1 and WIBR2 hESCs transfected with non-targeting siRNA (siNT). Top at right: Quantification of the gene expression of FUT1 in WT, siNT, and siFUT1, WA09 hESCs 30 h after FUT1 mRNA silencing, as measured by qPCR, and representative histograms showing relative counts of WT, siNT, and siFUT1, WA09 hESCs expressing α-fucose residues 54, 78, and 102 h after FUT1 mRNA silencing. Bottom: MFI of WT, siNT, and siFUT1, WA09 hESCs expressing α-fucose residues 54, 78, and 102 h after FUT1 mRNA silencing. (**B**) Schematic of Globo-H and SSEA-5 structures encompassing the α1,2-fucose end residue with corresponding GTs. (**C**) Top: Representative histograms showing relative counts of WT, siNT, and siFUT1, WA09 hESCs expressing SSEA-5 and MFI 54, 78, and 102 h after FUT1 mRNA silencing. Bottom: Representative histograms showing relative counts of WT, siNT, and siFUT1, WA09 hESCs expressing Globo-H and MFI 54, 78, and 102 h after FUT1 mRNA silencing. (**D**) Quantification of the gene expression of FUT2 in WT, siNT, and siFUT1, WA09 hESCs 30, 54, and 78 h after FUT1 mRNA silencing, as measured by qPCR. (**E**) Representative histograms showing relative counts of WT, siNT, and siFUT1, WA09 hESCs expressing HOPX and MFI 54, 78, and 102 h after silencing. $n = 4$ technical replicates. Data information: In (**A**, **C**, **E**), data are presented as means ± SD. Ordinary One-way ANOVA *$p < 0.05$, **$p < 0.01$, ***$p < 0.001$, ****$p < 0.0001$ or non-significant (NS). In (**D**), data are presented as means ± SEM. Two-tailed Student's t-tests *$p < 0.05$, **$p < 0.01$, ****$p < 0.0001$.

© The Author(s)

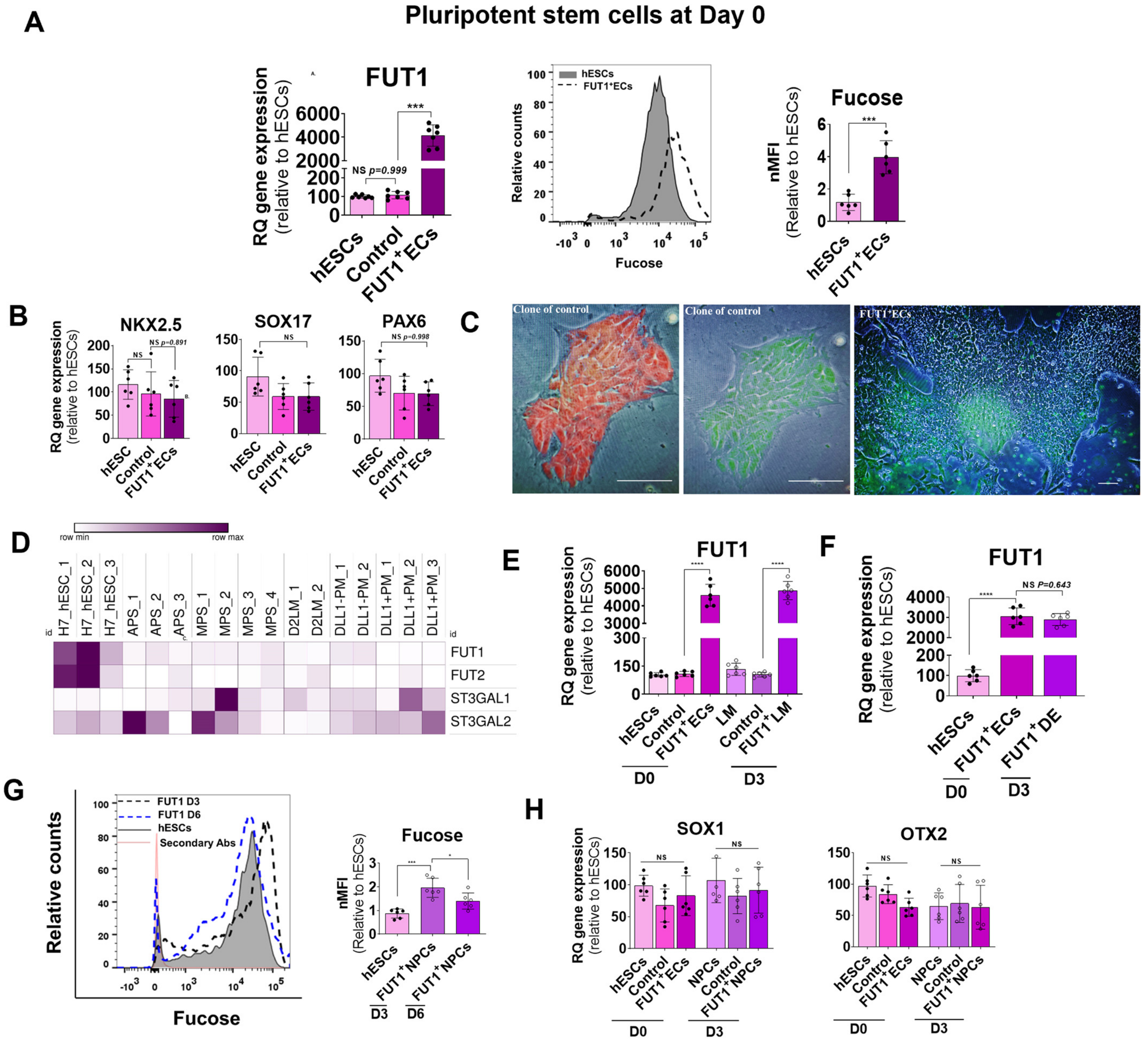

Figure EV4. Continuous expression of FUT1 amplifies the α-fucosyl glycoconjugates and impairs hESC differentiation.

(**A**) Left: Quantification of FUT1 transcripts in WT, mock-transfected (control ECs), and FUT1-transfected (FUT1$^+$ECs), WA09 hESCs in 3 clones for each line after transfection. Right: Representative histograms showing relative counts and MFI of WT hESCs and FUT1$^+$ECs expressing α-fucose. $n = 3$ technical replicates for each line. (**B**) Quantification of representative genes of the tri-germ layers, NKX2.5, SOX17, and PAX6, in WT hESCs and in 3 clones of control ECs and FUT1$^+$ECs for each line showing unchanged genes after transfection. (**C**) Left and center: Representative brightfield and endogenous fluorescence protein (red, left) and (green, center) images showing clone of control ECs 18 d after culturing sorted cells. Right: Representative bright-field and endogenous fluorescence protein (green) image showing clone of FUT1$^+$ECs 18 d after culturing sorted cells. Scale bars represent 100 µm. $n = 3$ technical replicates. (**D**) Heatmap of subset of RNA-seq-based gene expression profiles showing low expression of FUT1 1 d after H7 hESC differentiation into tissues that differentially expressed GT genes. Anterior primitive streak (APS), day 1 mid PS (MPS), and day 2 LM, PM, DLL$^-$, and DLL$^+$, PM cells. $n = 3$ per group. (**E**) Quantification of the mRNA expression levels of FUT1 in WT hESCs, control ECs, and FUT1$^+$ECs on day 0 and after differentiation into LM for 3 d showing high and constant expression of FUT1. (**F**) Quantification of the mRNA expression levels of FUT1 in WT hESCs and FUT1$^+$ECs on day 0 and after differentiation into DE for 3 d showing high and constant expression of FUT1. $n = 3$ technical replicates. Error bars represent ± SDs. (**G**) Representative histograms of relative counts and MFI of WT hESCs and FUT1$^+$ECs showing the expression of α-fucose residues in WT hESCs and FUT1$^+$ECs after differentiation into NPCs for 3 d and 6 d. (**H**) Quantification of NE markers showing SOX1 and OTX2 mRNA expression in WT hESCs, control ECs and FUT1$^+$ECs on day 0 and after differentiation into NPCs for 3 d. In all experiments, $n = 3$ technical replicates. Data information: In (**A**, **G**), data are presented as means ± SD. Ordinary One-way ANOVA $^{**}P < 0.01$, $^{***}P < 0.001$. In (**A**, **B**, **E**, **H**), data are presented as means ± SEM. Two-tailed Student's t-tests $^{****}p < 0.0001$ or non-significance (NS).

 © The Author(s)

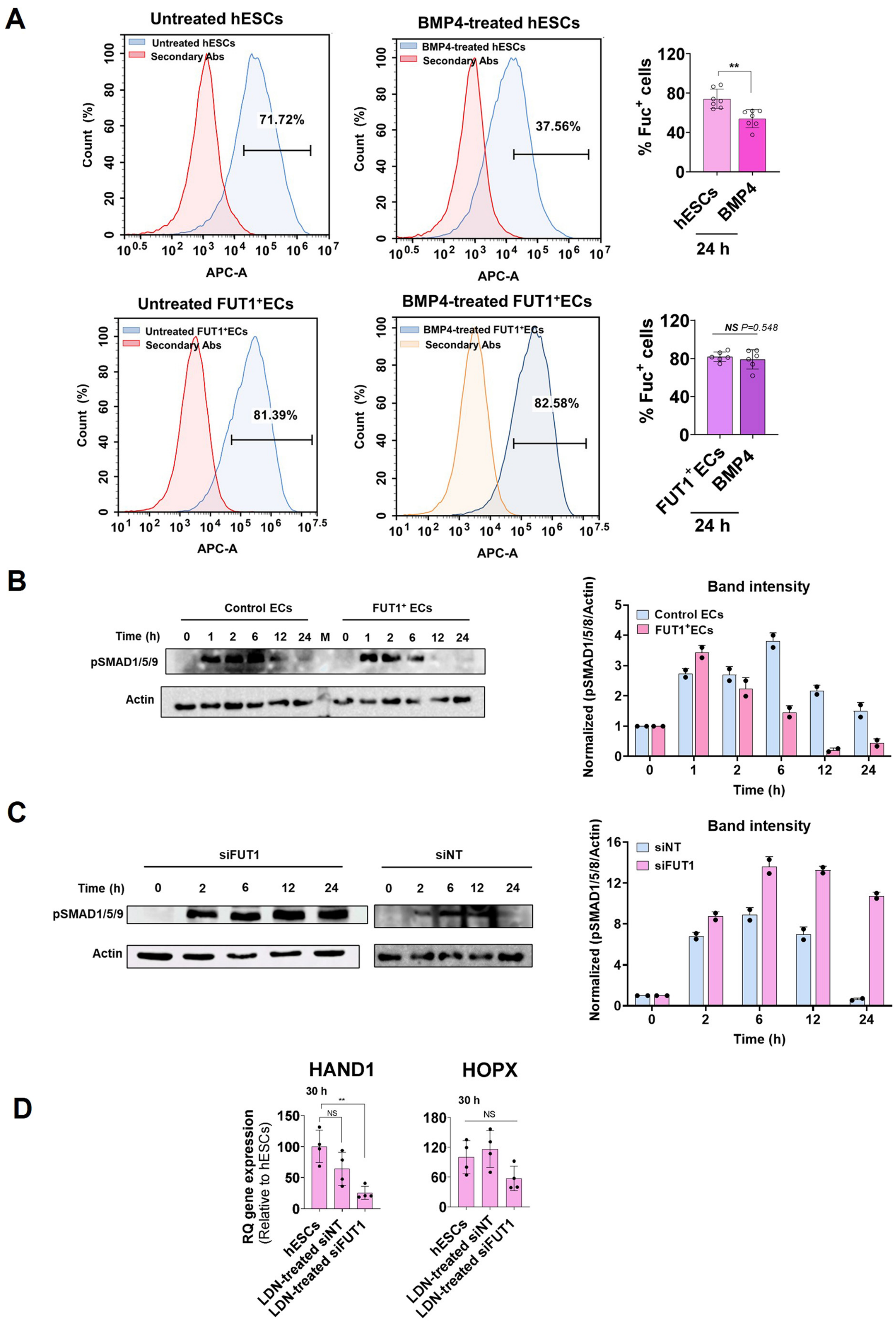
A
Untreated hESCs
Untreated hESCs
Secondary Abs
71.72%
Count (%)
APC-A
BMP4-treated hESCs
BMP4-treated hESCs
Secondary Abs
37.56%
Count (%)
APC-A
% Fuc+ cells
**
hESCs
BMP4
24 h
Untreated FUT1+ECs
Untreated FUT1+ECs
Secondary Abs
81.39%
Count (%)
APC-A
BMP4-treated FUT1+ECs
BMP4-treated FUT1+ECs
Secondary Abs
82.58%
Count (%)
APC-A
% Fuc+ cells
NS P=0.548
FUT1+ECs
BMP4
24 h
B
Control ECs
FUT1+ ECs
Time (h) 0 1 2 6 12 24 M 0 1 2 6 12 24
pSMAD1/5/9
Actin
Band intensity
Control ECs
FUT1+ECs
Normalized (pSMAD1/5/8/Actin)
Time (h)
C
siFUT1
siNT
Time (h) 0 2 6 12 24 0 2 6 12 24
pSMAD1/5/9
Actin
Band intensity
siNT
siFUT1
Normalized (pSMAD1/5/8/Actin)
Time (h)
D
HAND1
30 h
NS
**
RQ gene expression (Relative to hESCs)
hESCs
LDN-treated siNT
LDN-treated siFUT1
HOPX
30 h
NS
hESCs
LDN-treated siNT
LDN-treated siFUT1

Figure EV5. Constitutive expression of α-fucosyl end groups interferes with BMP signaling.

(**A**) Top left: Representative histograms showing relative counts of WT hESCs expressing α-fucose residues before and after BMP4 treatment for 24 h. Top right: Quantification of the percent of positive WT hESCs expressing α-fucose residues before and after BMP4 treatment for 24 h. $n = 3$ clones for each clone $n = 2$ technical replicates, Bottom left: Representative histograms showing relative counts of FUT1^{+}ECs expressing α-fucose residues before and after BMP4 treatment for 24 h. Bottom right: Quantification of the percent of positive FUT1^{+}ECs expressing α-fucose residues before and after BMP4 treatment for 24 h. $n = 3$ clones for each clone $n = 2$ technical replicates. (**B**) Left: Western blots of pSMAD1/5/8 and β-actin showing a time-course of Smad1/5/8 activity within FUT1^{+}ECs and control ECs after BMP4 stimulation for 24 h. Right: Quantification of the pSMAD1/5/8 intensities. The normalized values are relative to normalized β-actin. $n = 2$ technical replicates. (**C**) Left: Western blots of pSMAD1/5/8 and β-actin showing a time-course of Smad1/5/8 activity within silenced FUT1 and siNT cells after BMP4 stimulation for 24 h. Right: Quantification of the pSMAD1/5/8 intensities. The normalized values are relative to normalized β-actin. $n = 2$ technical replicates. (**D**) Quantification of HAND1 and HOPX 30 h after FUT1 silencing and 6 h after LDN193189 stimulation in WT, siNT, and siFUT1 hESCs relative to WT hESCs. $n = 4$ biological replicates. Data information: In (**A**, **D**), data are presented as means ± SD. Two-tailed Student's t-tests $^{**}p < 0.01$ or non-significance (NS).

 © The Author(s)
